# Is there no beauty in sexually dimorphic eyes? Facial attractiveness and White Europeans ocular morphology—Brief communication

**DOI:** 10.1371/journal.pone.0284079

**Published:** 2023-04-06

**Authors:** Dariusz P. Danel, Juan Olvido Perea-Garcia, Zdzisław Lewandowski, Anna Szala, Piotr Fedurek, Karel Kleisner, Sławomir Wacewicz

**Affiliations:** 1 Department of Anthropology, Ludwik Hirszfeld Institute of Immunology and Experimental Therapy, Polish Academy of Sciences, Wroclaw, Poland; 2 Institute of Psychology, Cognitive Psychology Unit, Leiden University, Leiden, The Netherlands; 3 Department of Human Biology, Faculty of Physiotherapy, Wroclaw University of Health and Sport Sciences, Wroclaw, Poland; 4 Center for Language Evolution Studies, Nicolaus Copernicus University, Toruń, Poland; 5 Department of Philosophy and History of Sciences, Faculty of Sciences, Charles University, Praha, Czech Republic; The Psychology Research Center (CIPSI), University of Minho, PORTUGAL

## Abstract

The link between human ocular morphology and attractiveness, especially in the context of its potential adaptive function, is an underexplored area of research. In our study, we examined the association between facial attractiveness and three sexually dimorphic measures of ocular morphology in White Europeans: the sclera size index, width-to-height ratio, and relative iris luminance. Sixty participants (30 women) assessed the attractiveness of the opposite-sex photographs of 50 men and 50 women. Our results show that in both men and women, none of the three measures was linked to the opposite sex ratings of facial attractiveness. We conclude that those ocular morphology measures may play a limited role in human mate preferences.

## Introduction

Compared to most other primate species, human ocular morphology stands out; our eyes are "wide and white" with unusually horizontally elongated eye fissures and uniformly pale sclera [1, 2]. On the intraspecific level, the peculiarity of human eyes is further reflected in sex differences regarding eye morphology observed, especially in Whites. For example, we have previously shown that White men have more horizontally elongated depigmented sclera surfaces [3, 4], while women had generally rounder eye fissures with a less pronounced contrast between irises and the surrounding sclera [4]. Similarly, Kramer and Russel [5] found that the sclera in White men is yellower and redder than in White women. Interestingly, despite the sexual differences regarding the shape and colour of the eye, the relative area of the externally visible sclera surface is similar in both sexes [4].

Sexual differences in the ocular features among Whites could be related to adaptive functions. Sexual selection and mate choice with locally specific aesthetic preferences, which have been argued to drive the emergence of sexually dimorphic characteristics [6, cf. 7], could be one of such functions [3, 4, cf. 8]. Therefore, dimorphic eye morphology may be a part of sexual ornamentation and a cue to one’s physical attractiveness. Indeed, several studies on scleral colouration showed that human faces with relatively yellower and redder sclerae are judged as less attractive [9–11]. Also, our previous study examined the link between ocular morphology and two biometric markers of facial attractiveness: facial averageness and sexual dimorphism [4]. None of the sexually dimorphic eye features, however, was linked to the facial attractiveness markers of interest, suggesting that the role of sexual selection in the evolution of eye morphology is questionable [4]. Nonetheless, our previous study did not directly test whether sexually dimorphic ocular morphology affects ratings of an individual’s facial attractiveness. Addressing this issue may provide greater credence to the notion that these sexually dimorphic features may not have evolved in the process of sexual selection. Here, we present results from our follow-up research, where we focused directly on facial attractiveness and examined whether sexually dimorphic eye features identified in our previous studies are related to the opposite-sex ratings of facial attractiveness. Drawing on our previous findings, we hypothesise that sexually dimorphic ocular morphology will not be related to the perceived facial attractiveness ratings in both sexes.

## Methods

### Facial portraits

We used the same 100 White European photographs from Czechia as in Danel [4]. The sample consisted of emotionally neutral facial portraits of 50 women (age: Mean ± SD = 23.64 ± 4.33, range: 19–36) and 50 men (age: Mean ± SD = 24.04 ± 3.92, range: 19–34) photographed according to standardised procedures. For more information, see *Methods* in Danel [4] and the citations therein.

### Eye measurements

We used the measurements of the eye features that we found sexually dimorphic in our previous studies [3, 4]. These included: i) the sclera size index (SSI), which is the ratio of the width of the exposed eyeball (the distance between the corners of the eye excluding *caruncula lacrimalis*) to the diameter of the iris; ii) the width to height ratio (WHR) of the eye outline; iii) the relative iris luminance (RIL) which reflected the relative contrast in luminance between the sclera and iris. The indices were calculated on averaged measurements obtained for the left and the right eye [for descriptive statistics and more information, see: 4].

### Ratings of the photographs

#### Participants

We recruited 60 (30 women) adult White Polish volunteers, all students at the Wroclaw University of Health and Sport Sciences. The mean age was M = 22.17 (SD = 2.408) for women and M = 20.13 (SD = 1.358) for men. We asked them to assess the physical attractiveness of the facial portraits. Raters of the opposite sex assessed the portraits: male faces were assessed by women, and female faces by men.

#### Study site

To prevent potential biases in attractiveness ratings resulting from using various uncalibrated displays with different brightness, colour temperature, size, and resolution, all rating sessions were conducted individually, at the same testing site, in the same room with diffused artificial overhead light and using the same computer equipment. Participants sat in front of a Dell Inspiron 5559 laptop with a 15.6" matte screen with a Full HD resolution (1,920 x 1,080 pixels) screen with stock (default) settings.

#### Rating procedure

An in-house computer application, Anthropologus [12], was used to display the stimuli. After brief instructions on the rating procedure, the facial portraits were presented in an order counterbalanced across the raters. The instruction (in Polish) was as follows: “In a moment you will see a series of facial portraits. Please rate the attractiveness of the people you see using the scale below the photo”. The rating procedure was self-paced; the exposure time of the facial images was not limited, while the pause time between consecutive stimuli (black screen) was 1000 ms. Using a mouse, subjects marked attractiveness ratings on the 7-point rating scale at the bottom of the screen, below the stimuli (1 indicated very unattractive and 7 indicated very attractive face). The ratings were collected individually from each participant (i.e., there were no group rating sessions).

### Statistical analysis

Data were analysed using a linear mixed-effects model (LMM) fit by the restricted maximum likelihood estimation (REML) with facial attractiveness rating score as a dependent variable, while SSI, WHR, and RIL were used as predictors. Because male facial attractiveness was assessed by women and vice versa, we constructed two separate LMMs for: i) male facial attractiveness (MFA) and ii) female facial attractiveness (FFA). The data of the predictors (i.e., fixed effects) were standardised (i.e., z-scored) before including in the model. Procedures regarding testing the model assumptions are provided in Supplementary Material. To control for the non-independence of data, Stimuli, as well as Participants (raters), were specified as a random intercept. This allowed us to account for the variability in attractiveness ratings coming from different Participants and Stimuli. Because in both models, the residuals were highly skewed (Shapiro-Wilk Normality Test for the MFA model W = 0.971, p < 0.001; the FFA model: W = 0.972, p < 0.001), the attractiveness scores were transformed before the LMM analysis using the Tukey Ladder of Power procedure, which applies a power transformation on a data set, using the ‘R companion’ package [13]. The transformation of the response variable resulted in the distribution of the residuals much closer to a normal range (MFA model: W = 0.997, p = 0.003; FFA model: W = 0.997, p = 0.018), while inspection of the qq-line showed little deviation from normality. To minimise the problem of collinearity, we first ran Kendal Tau correlations on all variable combinations to avoid including highly correlated variables (Kendal tau > 0.8) in the model. No correlation was greater than the cut-off value. We also calculated variance inflation factors (VIF) for all the variables, excluding any variables with VIF > 4. We calculated marginal (i.e., for fixed effects only) and conditional (i.e., for both fixed and random effects) R2 for the LMM model using the ‘lmerTest’ package [14] for R. Effect sizes for the predictors were estimated using the ‘effectsize’ package [15]. Additionally, with the ‘BayesFactor’ package [16], we obtained Bayes Factors (BF_01_) to investigate if our data support the null (i.e., no effect of sexually dimorphic ocular morphology on facial attractiveness ratings) or alternative (i.e., ocular morphology affects attractiveness ratings) hypothesis. LMM was performed using the ‘lme4’package [17] or R [18]. Data used in FFA and MFA models are in S1 and S2 Files, respectively. The analysis R-code is in S3 File.

### Ethics

Procedures performed in studies involving human participants were in accordance with the ethical standards of the institutional and/or national research committee (The Institutional Review Board of Charles University Faculty of Science; protocol ref. number: 06/2017) and with the 1964 Helsinki declaration and its later amendments or comparable ethical standards. All photographed individuals gave informed written consent to use their portraits in scientific research. All participants assessing the portraits informedly consented (“click-if-you-agree” consent in the computer application) to take part in the study.

## Results

All variables included both in the Male Facial Attractiveness (MFA), and Female Facial Attractiveness (FFA) models were weakly but significantly correlated with each other (Table 1), and VIF values were generally low (i.e., not exceeding 4; MFA model: RIL VIF = 1.15, SSI VIF = 1.18, WHR VIF = 1.03; FFA model: RIL VIF = 1.04, SSI VIF = 1.07, WHR VIF = 1.05). Therefore, all variables were included in the full models. The mean attractiveness ratings for individual men’s and women’s faces are provided in S4 File.

**Table 1:** Kendall correlation results between variables (z-scored) included in the models.

**Male Facial Attractiveness (MFA)**		
	**SSI**	**RIL**
**WHR**	tau = - 0.12; p < 0.001	tau = 0.04; p = 0.02
**RIL**	tau = - 0.23; p < 0.001	
**Female Facial Attractiveness (FFA)**		
	**SSI**	**RIL**
**WHR**	tau = -0.14, p < 0.001	tau = 0.12, p < 0.001
**RIL**	tau = 0.11, p < 0.001	

**Fig 1:**
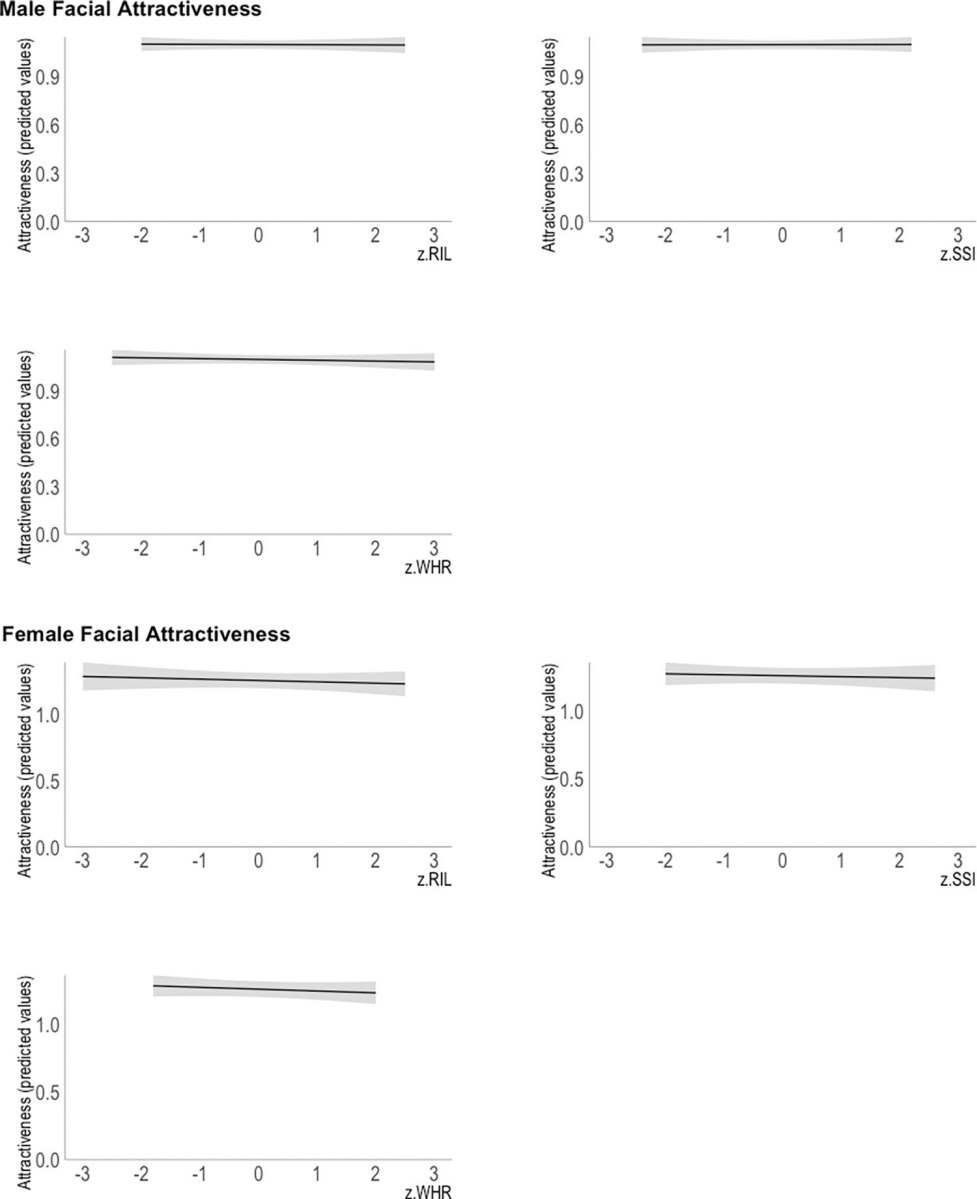
Relationship between transformed attractiveness (predicted values, adjusted for the means of other variables in the model) and z-scored (A) RIL, (B) SSI and (C) WHR in the male and female facial attractiveness model (MFA and FFA, respectively). The shaded area represents 95% CI.

Neither RIL, SSI, nor WHR was associated significantly with the opposite-sex attractiveness ratings for male and female portraits. Detailed results are presented in Tables 1 and 2 and Fig 1. Bayes Factors calculated for both MFA (BF_01_ = 28.10 ± 11.28%) and FFA (BF_01_ = 21.31 ± 10.94%) models provided strong evidence for no effect of sexually dimorphic ocular morphology on facial attractiveness ratings (i.e., our data were more likely under the null hypothesis compared to the alternative hypothesis).

**Table 2:** LMM results explaining attractiveness variance in the MFA and FFA models.

**Male Facial Attractiveness (MFA)**					
	**Estimate ± SE** ^**a**^	**t-value**	**p**	**η**_**p**_^**2**^ ^**b**^	**R** ^**2**^
**Intercept**	1.0961 ± 0.01398	78.396	< 0.001		Marginal: 0.0025
**RIL**	-0.0012 ± 0.0084	-0.143	0.887	4.26e-04	Conditional: 0.5851
**SSI**	0.0005 ± 0.0085	0.055	0.956	7.83e-05	
**WHR**	-0.0051 ± 0.0081	-0.634	0.529	8.55e-03	
**Female Facial Attractiveness (FFA)**					
	**Estimate ± SE** ^**a**^	**t-value**	**p**	**η**_**p**_^**2**^ ^**b**^	**R** ^**2**^
**Intercept**	1.2545 ± 0.0295	42.576	<0.001		Marginal: 0.0068
**RIL**	-0.0101 ± 0.0151	-0.671	0.506	9.66e-03	Conditional: 0.5987
**SSI**	-0.007 ± 0.0153	-0.454	0.652	4.38e-03	
**WHR**	-0.0136 ± 0.0152	-0.894	0.376	0.02	

^a^ SE–standard error

^b^ η_p_^2^ –partial eta-squared approximation [see: 15].

## Discussion

In general, our data show that in White European men and women, the morphological features of the eye that have been previously identified as sexually dimorphic are not linked to facial attractiveness. From a broad perspective, our results provide new insight into the role of human ocular morphology in the visual perception of facial attractiveness. Previous work on scleral characteristics showed that both colour (e.g., redness, yellowness) and brightness are a cue of individual attractiveness [11, 19, 20]. Similarly, studies on the limbal ring found that a darker and more distinctive annulus around the iris increases facial attractiveness in both men and women [21], and men with visible limbal rings are preferred as short-term partners [22]. More recently, Zhao et al. [23] found that in Asian women, larger elliptical areas between the eyelids are more common in female faces that are rated as more attractive.

The current study adds to this knowledge by showing that, in Whites, the ratings of facial attractiveness of men and women are not related to the measurements of the sexually dimorphic eye shape and contrast between the iris and the sclera. Thus, the examined dimorphic eye features may not be a part of the facial sexual ornamentation, and their role in attracting mates is limited. As such, the results of this study are in line with our previous research [3, 4], suggesting that sexual selection may not have been a significant force driving the evolution of the sexual dimorphism of the human eye in people of White European ancestry. Also, recently Caspar et al. [8] noted that drift (in conjunction with sexual selection but possibly also on its own) might explain interspecific differences in hominoid external eye appearance. Correspondingly, our data also suggest that other non-adaptive processes underlie the intraspecific differences between the sexes we inspected in this study. On the other hand, the apparent lack of association between the examined sexually dimorphic ocular morphology, and facial attractiveness may also result from the small morphological variation in the former, which is insufficient to affect individuals’ facial attraction. This hypothesis, however, should be addressed in future large-scale research involving various White populations.

As for the limitations, a considerable body of research shows that aesthetic preferences for facial attractiveness and sexual dimorphism may be conditional and influenced by a broad range of diverse factors (such as socioecological conditions: [24]; individual health status: [25]; cross-cultural settings: [26]; geographical variation in health and economic conditions [e.g. 27]. In our research, however, we have accounted for some of these factors by choosing a relatively homogenous sample of raters with a similar educational background and from the same geographical population (tertiary education students from Poland). Furthermore, using mixed effect modelling in the statistical analysis, we allowed for some variability in the ratings resulting from different participants and stimuli. However, examining the above-listed factors directly may provide new insight into the relationship between ocular morphology and attractiveness ratings. In addition, since the current follow-up study was conducted in the paradigm of sexual selection, we did not collect the same-sex ratings of the presented faces. Nonetheless, comparing the ratings provided by participants of various genders is a potential idea allowing for a more general analysis of aesthetic preferences towards sexually dimorphic eye features.

In conclusion, in the current study, we examined the association between an individual’s facial attractiveness and three sexually dimorphic measures of ocular morphology in White Europeans. Both in men and women, neither eye fissure shape nor the relative size of the exposed sclera nor the relative luminosity of the iris were linked to the opposite sex ratings of facial attractiveness. We conclude that the analysed traits play a limited role in human mate preferences. Thus, sexual selection may not have been a major force driving the evolution of the sex differences in Whites’ ocular morphology.

## Supporting information

S1 File Data supporting FFA model. (CSV)

S2 File Data supporting MFA model. (CSV)

S3 File R-code used for data analysis. (R)

S4 File Means (and standard deviations; SD) of individual female and male faces. (DOCX)
