# Supplementary material for: Is there no beauty in sexually dimorphic eyes? Facial attractiveness and White Europeans ocular morphology—Brief communication
Source: PLoS One. 2023 Apr 6;18(4):e0284079. doi: 10.1371/journal.pone.0284079 (PMC10079083; doi:10.1371/journal.pone.0284079)
Supplement: S4 File — (DOCX) [file pone.0284079.s004.docx]

**Table S1. Means (and standard deviations; SD) of individual female faces.**

| **Photo id** | **Mean (SD)** | **Photo id** | **Mean (SD)** | **Photo id** | **Mean (SD)** | **Photo id** | **Mean (SD)** | **Photo id** | **Mean (SD)** |
| --- | --- | --- | --- | --- | --- | --- | --- | --- | --- |
| 2040 | 1.33 (0.71) | 7060 | 1.47 (0.68) | 8070 | 2.10 (1.03) | 9023 | 1.43 (0.63) | 9060 | 2.47 (1.36) |
| 4050 | 1.37 (0.72) | 7070 | 1.87 (0.86) | 8080 | 1.33 (0.84) | 9030 | 1.97 (1.07) | 9062 | 2.23 (1.16) |
| 4090 | 2.30 (1.24) | 7090 | 1.57 (0.86) | 8090 | 2.53 (1.25) | 9032 | 1.57 (0.86) | 9063 | 2.97 (1.27) |
| 4120 | 1.47 (0.73) | 7100 | 2.77 (1.19) | 8100 | 1.60 (0.85) | 9033 | 1.80 (0.96) | 9070 | 1.27 (0.52) |
| 4130 | 1.33 (0.55) | 8010 | 2.40 (1.33) | 8110 | 1.37 (0.56) | 9040 | 1.43 (0.68) | 9072 | 3.77 (1.75) |
| 4170 | 1.93 (1.20) | 8020 | 1.57 (0.90) | 9010 | 1.83 (1.18) | 9042 | 1.40 (0.84) | 9073 | 1.87 (1.11) |
| 4200 | 1.93 (1.23) | 8030 | 1.40 (0.62) | 9012 | 1.27  (0.52) | 9043 | 1.70 (1.02) | 9082 | 2.23 (0.93) |
| 6060 | 1.47 (0.73) | 8040 | 1.53 (0.78) | 9013 | 3.67 (1.32) | 9050 | 2.07 (1.11) | 9083 | 1.60 (0.85) |
| 6080 | 1.83 (1.23) | 8050 | 1.53 (0.73) | 9020 | 2.33 (1.32) | 9052 | 3.23 (1.72) | 9102 | 1.77 (0.93) |
| 7040 | 1.80 (0.76) | 8060 | 2.97 (1.54) | 9022 | 1.73 (0.87) | 9053 | 2.77 (1.43) | 9112 | 1.33 (0.61) |

**Table S2. Means (and standard deviations; SD) of individual male faces.**

| **Photo id** | **Mean (sd)** | **Photo id** | **Mean (sd)** | **Photo id** | **Mean (sd)** | **Photo id** | **Mean (sd)** | **Photo id** | **Mean (sd)** |
| --- | --- | --- | --- | --- | --- | --- | --- | --- | --- |
| 1011 | 2.33 (1.27) | 4021 | 1.67 (0.84) | 4161 | 1.47 (0.78) | 5041 | 2.37 (1.45) | 5161 | 2.30 (1.42) |
| 1041 | 1.37 (0.61) | 4031 | 2.03 (1.19) | 4191 | 3.87 (1.76) | 5051 | 2.17 (0.95) | 5171 | 3.63 (1.71) |
| 1051 | 4.17 (2.00) | 4051 | 2.60 (1.28) | 4211 | 2.10 (0.99) | 5061 | 2.00 (0.98) | 5181 | 3.07 (1.74) |
| 1061 | 2.40 (1.248) | 4061 | 3.57 (1.67) | 4221 | 2.63 (1.24) | 5071 | 3.00 (1.60) | 5191 | 2.13 (1.28) |
| 2011 | 1.83 (1.15) | 4071 | 2.17 (1.15) | 4231 | 2.20 (1.24) | 5091 | 1.77 (0.97) | 5211 | 2.70 (1.39) |
| 2021 | 1.40 (0.72) | 4081 | 1.83  (1.02) | 4241 | 1.97 (1.03) | 5101 | 1.67 (0.96) | 5231 | 2.93 (1.70) |
| 2031 | 1.53 (0.78) | 4101 | 2.83 (1.34) | 4251 | 2.10 (1.30) | 5111 | 2.47 (1.31) | 6051 | 3.33 (1.67) |
| 2051 | 1.87 (0.78) | 4111 | 3.27 (1.28) | 5011 | 1.90 (0.96) | 5121 | 3.37 (1.52) | 6091 | 1.93 (0.98) |
| 3011 | 2.33 (1.09) | 4141 | 2.47 (1.20) | 5021 | 2.43 (1.16) | 5141 | 2.27 (1.31) | 6121 | 2.47 (1.17) |
| 3021 | 1.27 (0.52) | 4151 | 2.83 (1.39) | 5031 | 2.17 (1.18) | 5151 | 2.10 (1.09) | 6131 | 3.40 (1.9) |
